# A Real-World Single-Centre Study of Patients with Diabetic Macular Oedema Who Wore a Home-Use Sleep Mask (Noctura 400) for One Year

**DOI:** 10.1155/2021/6612126

**Published:** 2021-06-15

**Authors:** Ulrich Meyer-Bothling, Oliver Meyer-Bothling, Marika Pinney

**Affiliations:** Ophthalmology Department, Ashford and St Peter's Hospital NHS Foundation Trust, London Road, Ashford TW15 3AA, UK

## Abstract

A “Real-World” single-centre observational study was carried out to analyse the effects of enhanced patient interaction with the use of the Noctura 400 sleep mask on a group of 26 diabetics displaying diabetic retinopathy (DR) and diabetic macular oedema (DMO), of which 24 completed the study. We hoped to find if patient compliance could be maintained and to determine the anatomical and functional consequences of consistent mask wear. While this study was ongoing, COVID-19 impacted on normal eye clinic practice, allowing an unexpected analysis of the effects of this disruption to the clinical system on mask wear and disease progress. Throughout the whole study, outcomes were positive, with a high level of consistent patient use of the mask, above 74% up to and beyond 1 year. Even during the COVID-19 1st lockdown in England, the patients maintained a 65% nightly light mask compliance. Statistically significant improvements in maculopathy, including cyst reduction (56.4% of eyes with cysts exhibited cyst shrinkage), and visual acuity (VA) improvement (42/48 eyes gained 5 letters or more) were observed and maintained to the end of the study. Anatomical improvement or stability was recorded in all but one study eye. This investigation shows that given that there is appropriate interaction with patients who are self-treating in home environment, a high level of patient compliance can be maintained, even while there are disruptions to the normal hospital clinic setup.

## 1. Introduction

An important complication of diabetes is DR, a major cause of world blindness [1]. In an attempt to reduce the burden, some countries are establishing or have established screening programs such as the one currently in the UK [2]. Even where screening is not in place, the trend is towards earlier detection of diabetic retinal and macular changes prior to the onset of sight-threatening disease. A key sight-threatening event in diabetic eye disease is the development of DMO. Central to the development of DMO are the three main mediators, linked to chronic hyperglycemia, which are inflammation, production of reactive oxygen species, and continued hypoxia [3]. They combine to elevate a range of vasoactive cytokines such as vascular endothelial growth factor (VEGF) producing the pathological vascular changes that result in blood-aqueous barrier breakdown and increased retinal vessel permeability [3, 4]. It is important to note that DR and DMO are not just the product of vascular tissue pathology but the consequence of changes to the neurovascular unit as a whole. The concept of the retinal capillaries plus the surrounding glia and neurones acting in concert to control fluid transport and metabolic transfer is providing us with a more complex understanding of the unfolding pathologic events in the target tissue [3, 5].

In recent decades, the traditional lasering of microaneurysms and areas of thickening in a grid pattern treatment has been successfully augmented or superseded by intraocular antivascular endothelial growth factor (anti-VEGF) injections to reduce macular oedema, decrease vessel permeability, and prevent blood aqueous barrier BAB breakdown [3–7]. In addition, various types of intraocular slow-release steroid implants are now in use, focussing on the fact that chronic inflammation is a key contributory factor to the development of DR and DMO. Increased leukostasis with upregulation of ICAM-1 is an early inflammatory event while later monocyte/macrophage infiltration of vessels and then into neural tissue is promoted by massively increased amounts of retinal and vitreal MCP-1 [3]. These anti-VEGF and anti-inflammatory treatments have revolutionised the care of sight-threatening DR and DMO [6, 7]. Indeed, a broader approach to chemokine and cytokine inhibition is under close scrutiny for a future direction [3] as is intravitreal human stem cell delivery and induction of neuroprotective growth factors to combat DR [8].

Clearly, the currently established, and future potential, treatments are essentially clinic- and hospital-based. In addition for those patients with morphological, but as yet little or no visual evidence of progression, treatment is observational and to encourage good metabolic control of not only HbA1c but also blood pressure and LDL cholesterol levels [9]. Yet early pathology is there because we are now beginning to appreciate by coherence tomography angiography that diabetic patients even with no apparent evidence of DR show clear signs of microvessel alteration in the macula [10]. Further, among recent trials, there are those examining the efficacy of anti-VEGF for patients who up to now would not normally qualify for intraocular injection [11, 12]. Although it appears that DMO patients with good central vision gain little functional benefit from early intervention, being either photocoagulation or anti-VEGF [11], there may be a better case for anatomic gain especially in those with slightly more advanced pathology [12]. Thus, the value of treating patients at the cusp of typical intervention with modest visual loss and macular pathology is under scrutiny.

By way of contrast, the visual healthcare budgets and eye department patient burden of many countries have been stretched by the success and rapid expansion of anti-VEGF injection therapy for DMO [11, 13] and other interventions [13]. It is also the case that anti-VEGF injections and steroid implants are not without serious side effects [14] and some level of patient stress [15]. There is, therefore, a need for earlier treatments of limited or no risk that preferably can be moved away from the hospital clinic to the home especially in these times of COVID-19 outbreaks [16].

Prior to the development of DR and DMO, there are preclinical changes to microvessels [10] accompanied by retinal neural and glial reactions [17, 18]. As the pathology progresses, key generators are hypoxia and VEGF production both of which drive new vessel formation in the normal developing retina [19]. On the other hand, they, along with inflammation and oxidative stress, compromise the neurovascular unit of the hyperglycemic retina [3, 20]. The question arises whether decreasing hypoxia is a possible therapeutic direction. That would seem to be the case because supplemental oxygen through the nasal cavity for 3 months improved DMO by reducing macular thickness [21]. Certainly, this and other similar oxygenation studies provide reasonable proof of principle but they are hardly a practical therapeutic option. Light therapy however may offer a viable alternative antihypoxia solution.

It is known that the massive oxygen usage by rods during dark adaption presents a strain not only on the choroidal circulation but also on the retinal vessels. Conversely, in daylight, the oxygen consumption by the outer retina drops considerably, found to be as much as halved in some species [22]. Further, it is believed that the choroid supplies only one-quarter of the thickness of the dark-adapted retina, increasing to half the retinal thickness in the light [22]. While retinal vessels contribute to only around 10% of the oxygen needed in the dark-adapted outer retina, in the light, their contribution can be negligible [23]. Despite this low contribution to the oxygen requirement, these retinal vessels are compromised during and even before DR and DMO development and are both metabolically and functionally taxed, particularly under fully dark-adapted conditions [20, 24]. Logically, a clear treatment option is to prevent dark adaption during sleep (the only time we truly dark adapt in the modern well-lit world) and thus relieve demand on the compromised inner retinal vessels and prevent prolonged periods of local hypoxia [24, 25]. A practical option is gentle illumination of the retinal rods by means of a sleep mask [24].

Only one such sleep mask designed to emit green light into the eyes through the closed lids of sleeping patients has been marketed to date, Noctura 400 (PolyPhotonix, Sedgefield, UK). Initial trials demonstrated both safety and a level of efficacy [26, 27] but in the subsequent CLEOPATRA phase III two-year trial [28], very poor patient compliance with mask wear contributed heavily to the failure to demonstrate further efficacy. Unfortunately, this has led to questioning the principle of oxygen sparing as a realistic therapeutic direction for diabetic eye disease and/or whether Noctura 400 is a satisfactory means of delivering that therapy [28, 29]. Essentially, is the principle flawed or is the delivery mechanism, in the form of Noctura 400, not fit for purpose? A failing of both, either one, or neither needs careful consideration.

As the authors of the CLEOPATRA trial publication [28] state “further studies should include additional interventions to increase patient engagement in wearing the light masks to evaluate whether this intervention is sustainable over the lifetime of their diabetic eye disease.”

A “Real-World” single-centre investigation was undertaken on patients who were given Noctura 400 for home use. This study included additional patient engagement and examined whether the patient compliance and experience with the device could be improved. Furthermore, if there was good Noctura 400 wear, what were the anatomic and functional consequences?

We consider that a noninvasive home therapy would be a huge benefit to DR and DMO treatment going forward especially for patients with relatively early macular pathology, not yet requiring invasive therapy.

## 2. Materials and Methods

### 2.1. The Device

**Figure 1:**
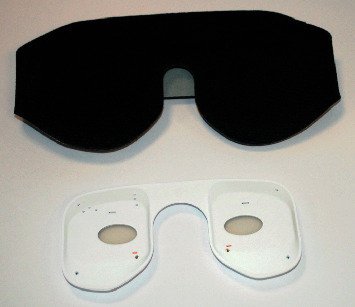
Picture of the black fabric mask and the white plastic pod housing the OLED lighting that make up Noctura 400.

The present observational study was undertaken to evaluate the usefulness of Noctura 400 in the treatment of DR and DMO in a UK National Health Service (NHS) setting. Noctura 400 (PolyPhotonix, Sedgefield, UK) is a CE-marked sleep mask that consists of a fabric mask that houses a plastic pod (Figure 1). The pod contains organic light-emitting diodes (OLEDs) that deliver a glow of predominantly green light (the OLED spectrum had a peak at 502 ± 5 nm) through closed lids to the retina while the patient is sleeping. The selected wavelength distribution was chosen to match the scotopic response curve for activation of rods. The light reaching the retina has been calculated to be 2 scotopic Trolands and sufficient to prevent full dark adaption [30].

Capacitive sensors activate the OLEDs in the pod to deliver the appropriate light dose for a maximum of 8 hours. These sensors require close facial contact to record mask wear. Every two seconds, the mask detects if it is being worn or not and stores this in an onboard memory chip. This data can be downloaded at an appropriate time, thus providing an effective and accurate mechanism to record the mask-wearing habits of each patient. At the end of the 3-month mask life, the whole unit consisting of fabric mask and pod was replaced.

The old pods were sent to the reading centre for 3-month determination of mask wear. The data was downloaded wirelessly, using RFID technology. A unique identifier on each mask linked each pod to each patient, ensuring the accurate matching of the pod data and patient while maintaining anonymity.

**Figure 2:**
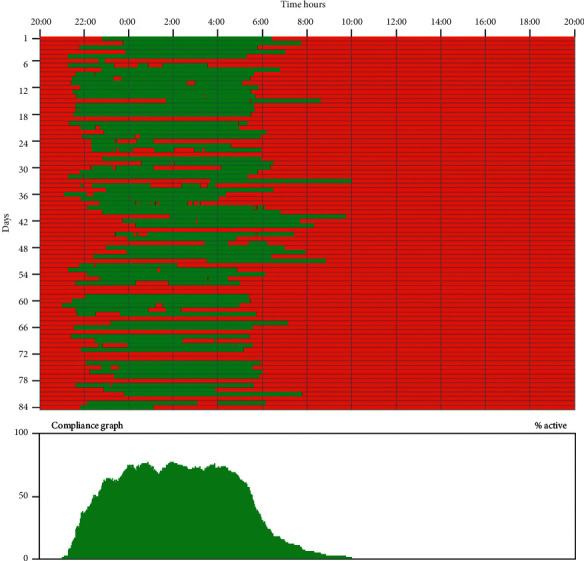
The mask wear information from a patient for a 3-month period. The nightly mask wear is shown as a series of horizontal green lines whose position and length tell us when and how long the patient wore the device.

The operational used data consists of “on” and “off” information at two-minute intervals throughout the nightly operational period. Downloading the collected data gives the team a measurement of nightly mask wear for each patient. A graphical presentation of such data can be seen in Figure 2. Thereafter, a reasonable estimate of percentage compliance with treatment can be determined for each patient from their average daily mask wear against either the estimated reported hours of sleep or the programmed 8 hours of therapeutic light available each day.

### 2.2. The Patients and the Investigation

The study complied with the Declaration of Helsinki and was supported and funded through the UK Research and Innovation (UKRI) funding scheme for innovative medical technologies, grant number 105341. A total of 26 patients (Table 1) with DR and mild DMO were recruited for the study and they were followed for the 13 plus-month duration of investigation. All subjects were required to be over 18 years of age and pregnancy was an exclusion criterion. In addition, the patients needed to have a central macular thickness of less than 400 *μ*m (therefore, not qualifying for standard therapeutic anti-VEGF intervention in the UK, as defined by the National Institute of Clinical Excellence (NICE)).

Six of the 26 DMO patients had previous interventions (4 had laser and 2 anti-VEGF injection) but these interventions ended not less than 6 months prior to recruitment. It was decided that any patients' participation whose DR or DMO status deteriorated to the point where they required laser or intravitreal injection would end.

### 2.3. Patient Evaluations

All patients were due to attend hospital visits at regular intervals that included a baseline visit (visit 1) plus up to 4 further visits at approximately 3-month intervals. The patients underwent an initial clinical assessment at baseline (visit 1) that involved assessing individual diabetic status (HbA1c), and general medical history was noted as was their ocular history and current status. These were assessed again at each visit up to and including their exit visit (scheduled to be completed at 12 months but actually took 13 or more months; see COVID-19).

At baseline (visit 1) and at all 4 subsequent visits, there was a detailed fundus examination with dilated pupils of the retina and colour fundus photography. Optical coherence tomography (OCT) (Heidelberg Spectralis, Germany) was conducted on the maculae of both eyes. The presence, size, and location of macular cysts and fluid accumulations at both maculae and in subretinal positions were recorded. Macular volume, central maximum thickness, individual thickness in all 9 zones, thickness in inner versus outer zones, and central subfield thickness (CST) were determined and subsequently analysed. Grading of the patients' retinal and macular disease statuses was based on national screening programme guidelines [2, 31].

During the ophthalmological examination VA, LogMAR ETDRS (Early Treatment Diabetic Retinopathy Study) and Pinhole VA were measured for each eye of all participating patients. Whichever measurement was higher for a given patient, we labelled “Best VA.” In addition, contrast sensitivity was determined (Pelli-Robson chart). The various visual assessments were conducted at each visit.

Adverse events and their duration were noted in the patients' log following visit 1 onwards until the end of the study. Thereafter, they were reviewed and separated into likely/possible/unlikely to be related or unrelated to Noctura 400 nightly wear. Particular attention was paid to events that might be mask-related such as discomfort, light-related disturbance, or wakefulness. All patients were interviewed in clinic to assess their sleep record and also to identify adverse sleep disturbance to permit accurate compliance calculation according to the formula:

(1) $$\begin{array}{c} \left(\frac{T_{t}\;}{\left(T_{r}\times \;D\right)}\right)\times 100=\%\text{ compliance}, \end{array}$$

where *T*_*t*_is the total time the mask was worn, *T*_*r*_is the reported hours of sleep per night, and *D* is the number of days the mask could have been worn.

In addition, the patients filled in a Pittsburgh Sleep Quality Index (PSQI) questionnaire at each of their visits.

### 2.4. Patient Noctura 400 Instruction and Support

We considered that, for Noctura 400 to have the most efficacious benefit through optimised use, patient education and support were essential from the outset. It was equally important that the patient be comfortable with their therapeutic device and able to identify personally with their home treatment in a voluntary and active manner [32].

At baseline (visit 1) each patient was introduced to Noctura 400 with a careful explanation of how it operates and how it is worn at an in-depth one-to-one session. During the patient introduction, the patient was able to try on a demonstration mask, activate it, and experience the light. The science behind the mask was discussed, mask wear recording explained, and optimum mask storage outlined. A patient pack containing point-by-point information reinforced the session and included Noctura 400 instructions and a detailed troubleshooting guide. Emphasis was placed on careful reporting by the patients of their sleep hours.

Phone contact was established with each of our patients so that follow-up calls and continuous additional support could be maintained during the 3 months or so between hospital visits. The patient/clinic follow-up phone call schedule was instigated. Routinely 3 to 4 days after each mask start date, there was a check-up to make sure that the mask was delivered and operational, there were no user problems, and Noctura 400 was being worn nightly. At around 10 days after the first mask start date, a mask wear check-up phone call was made, and any feedback was addressed. At and beyond 5 weeks, a call and text provided further support, encouragement, and information. A week prior to clinic visit, the patient was called or sent a text message and provided with information about the forthcoming visit and reminded to bring the mask to clinic.

A patient satisfaction survey was conducted at the end of the study (reported elsewhere).

### 2.5. COVID-19

The viral pandemic in general and specifically lockdowns to reduce viral spread and hospital personnel relocation to other duties inevitably was (and is) disruptive to many ongoing trials worldwide including this one. However, none of our trial patients was reported as being COVID-19 positive up to the trial end and all patients were encouraged to continue mask wear as normal even if their clinic visit was cancelled or altered. Additional patient telephone contact and follow-up were instigated during this period to ensure seamless treatment delivery and patient support.

In England, the first national COVID-19 lockdown began on 23 March 2020 and started to ease by 28 May 2020. The lockdown meant that, for the recruits, the final visit was extended out from 12 months to 13 months or more. However, the disruption did give us the opportunity to evaluate device use during the lockdown period.

### 2.6. Statistical Methodology

The data unit was patients' eyes. Initially, descriptive statistics, paired *t*-tests, and signed-rank tests were calculated to assess the differences between the first and last visit of each eye in all outcomes. Waterfall plots were used to evaluate visually the overall eye performance for each outcome. These graphs show the differences between the last visit and the baseline for every eye sorted by magnitude.

## 3. Results

### 3.1. Patients

A total of 26 patients were recruited to this study (Table 1) and of these, 24 reached their exit visit (92.3%). One patient withdrew because of the development of unrelated severe systemic health issues. The second dropped out of contact following visit 2 and submitted a virtually unused mask over the first 3 month period. Given the “Real-World” nature of the study, patients were encouraged to carry on with Noctura 400 treatment after exit. The HbA1c at baseline had a median value of 62.5 and fell to 58.0 by the trial end. During COVID-19 lockdown the median value was 56.0 (Table 1).

From the patient log, 27 adverse events were recorded over the period of the trial and no major Noctura 400-related problems were identified. Twelve events were unrelated, eight were unlikely to be related, six were possibly related events (ranging from periodic watery eyes and temporary itchiness of the face to mild ocular redness, occasional blurring, some temporary sore eye issues, and transient headaches in the morning for a few days), and one was definitely mask-related event (irritation of the bridge of the nose). There were no light-related adverse events. However, one patient with some preexisting minor sleep issues found that they got worse up to visit 2 (3 months of mask wear), thereafter, for the rest of the investigation up to their exit visit, sleep was reported as being normal.

### 3.2. Mask Wear and Compliance

**Figure 3:**
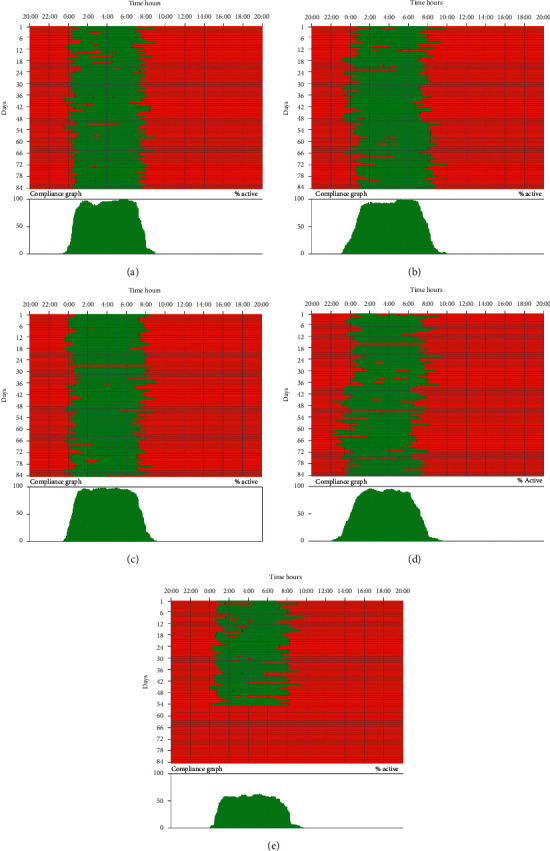
The study mask wear data from a patient who completed mask wear for over 1 year. It can be seen from this patient that mask wear is high throughout and it was in line with the patient-reported sleep hours making compliance high and consistent. Note that this patient required an additional 5th mask because of clinic delay of exit visit due to COVID-19.

Mask wear was calculated from the integrated mask face adherence software recordings and self-reported sleep hours. This sensor and software gave us a night-time determination of mask contact with the face (Figures 2and 3) per patient for each visit. Patient-reported sleep hours provided an average estimate of nightly sleep so that compliance could be calculated from the two (see Materials and Methods). It can be seen that the median compliance at the 3rd-month visit (visit 2) was over 80%, fell a little thereafter, but was over 76% by the exit visit (Table 2). Indeed, some patients reached the best individual sleep mask compliance in this study before and at exit visit (Figure 3). There was no significant change in compliance across the study (*p*value = 0.3048).

At the onset of COVID-19 and 1st lockdown, some reduction in sleep hours was noted, since reported sleep dropped from an average of 7.1 hours per night to 6.7 hours. Patients continued to wear the mask during lockdown but compliance dropped to just a little over 65%. Examination of the range revealed a widening during lockdown compared to the prelockdown range. Even so the range did not drop below 50% compliance while others were over the 80% mark (Table 2).

### 3.3. Anatomic Change

**Figure 4:**
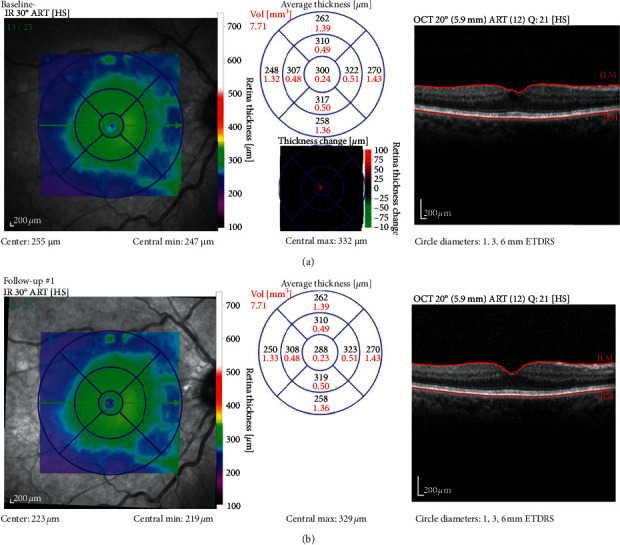
OCT of the macula from the right eye of a patient at baseline and at the 1st visit following 3 months of mask wear. The topographical map is reasonably flat at baseline and remains so throughout (shown only at 2nd visit). The central cross-sectional image (top right) shows a circular pocket of hypofluorescence in the foveal region. This cyst is resolved at the 2nd (3 months) visit (bottom right) and remains so up to and including the exit visit.

Clinical assessment of retinopathy and maculopathy, based on fundus examination and OCT (Figure 4), showed that in 2 patients both eyes improved, in 11 patients one eye improved while the other remained stable, 10 patients remained stable in both eyes, and in the final patient one eye improved but the other developed proliferative diabetic retinopathy (PDR). Thus, 16/48 eyes (33.3%) improved, 31/48 eyes (64.6%) were the same at study end as they were at baseline, and only 1/48 eyes (2.1%) deteriorated.

Foveal and parafoveal cysts were recognized by OCT as hypofluorescent structures from around 10 *μ*m to 100 s of *μ*m in diameter (Figure 4). Cysts of various sizes were identified in 39 of the 48 eyes (from the 24 completing patients) during the baseline examination. Cysts started to resolve at 3 months (Figure 4), while by the exit visit, all evidence of cysts was eliminated from 3 maculae and there was clear cyst reduction in numbers and/or size (but not total elimination) from a further 19 subjects. Thus, for 22/39 eyes (56.4%), their pathological status, with respect to cysts, improved over the course of the trial. A further 16 showed no detectable difference in cyst status at the end of the investigation when compared to the outset. In only one eye, the one with worsening maculopathy and PDR did large macular cysts present at study end.

Central maximum thickness did not show any statistically significant change, whereas macular volume showed a modest but significant decrease of 0.08 *μ*m^2^ from baseline (*p*value = 0.0023) (Table 3) that became apparent 9 months into the study. In the 48 eyes, all but 1 of the 9 zones, there was a small mean thinning over the study period, though standard deviations (SDs) were extremely large such that thinning was only marginally significant in 3/9 zones (*p*value < 0.05) and highly significant in 1/9 zones (*p*value = 0.002). The improvement of these outcome variables was first seen during the visit at 6 months and remained constant thereafter. However, if the best improvement from all zones was selected, then a mean decrease of over 13 *μ*m was observed (*p*value < 0.0001) (Table 3).

**Figure 5:**
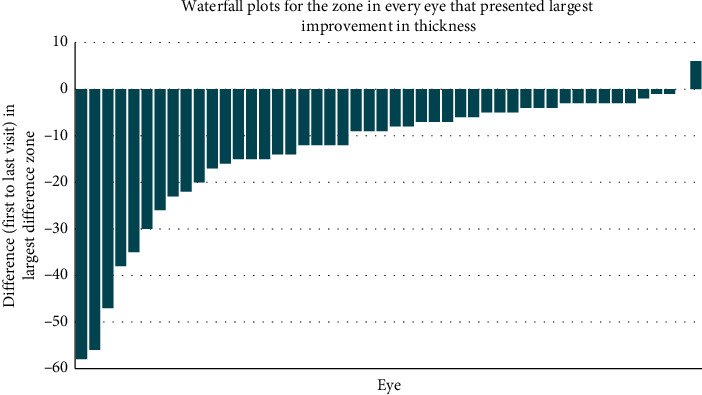
The waterfall plot shows the largest improvement irrespective of zone for each eye of the patients completing the study. Only 1 eye deteriorates in every zone. The vast majority showed some level of thinning (46/48). Some eyes that show marked improvement in their best zone (10/48) show 20 *μ*m thinning or better.

Waterfall plots were constructed to illustrate focal changes by OCT in the macula and its various zones. Eyes from the patients are sorted from the smallest to the largest difference between the baseline and exit visit for each parameter. Overall, the macula seemed to improve in more than 50% of the patients albeit by a small amount. Looking at the specific waterfall plot (Figure 5) for the largest positive change irrespective of zone, only 1 of the 48 maculae had only deterioration in every zone. The rest (21/47 had their best zone thinning in excess of 10 *μ*m while 5/47 had their best zones thinning by 30 *μ*m or more. The vast majority showed some level of thinning (96%) of which a few showed a marked level of thinning (20.8%) during the study period.

### 3.4. Functional Change

**Figure 6:**
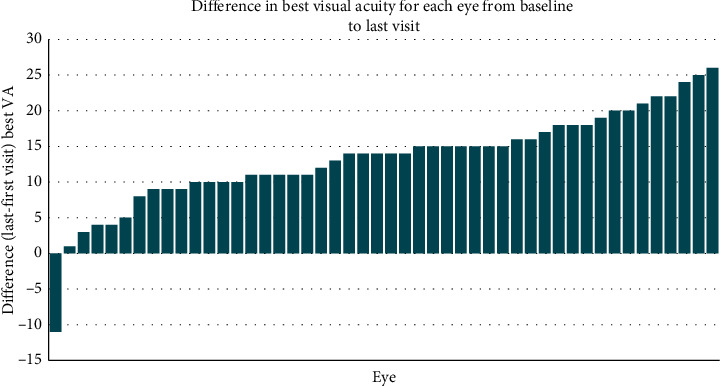
The waterfall plot showing differences from baseline to study end in best VA for each eye in the investigation. Only 1 is below the line, while the rest show some acuity improvement such that 42 of the 48 eyes have an improvement of 5 letters or more.

The majority of patients showed functional improvement based on VA. Again waterfall plots were constructed to show change in acuity over the study period from baseline to exit for the 48 eyes of 24 patients who completed the study. These waterfall plots show that almost all patients improved their acuity measures such that VA deteriorated in 1 eye but improved in 47 (Figure 6). For 42/48 eyes (87.5%), the improvement was in excess of 5 letters, while for 15/48 eyes (31.2%), the improvement was 15 letters or more. The mean and SD for improvement comparing baseline versus exit visit was highly significant by both paired *t*-test and signed-rank test with a mean best VA improvement of 12.4 ± 7.2 letters and a median of 14 (Table 4). Contrast sensitivity did not show any statistically significant change throughout the study.

## 4. Discussion

This “Real-World” single-centre observational study was carried out to analyse the effects of the use of the Noctura 400 sleep mask on a group of people with diabetes displaying DR and some DMO, in order to evaluate the consequences of consistent mask wear on macular pathology, VA, and contrast sensitivity. The present investigation had a low incidence of adverse events associated with mask wear. Only one was definitely mask-related but there were 6 other possibles. It was very encouraging that, in the whole year, only two dropped out and for events unrelated to the mask.

There was no control group in this study; also, the number of patients for the study conducted was limited; however, there were clear anatomic and functional improvements among mask wearers. The DR diagnosis date was not available to evaluate which patients were more likely to show improvement depending on how long they have had the condition. The fact that 13 out of 24 patients improved their retinopathy and maculopathy in at least one eye over the duration of the study was gratifying as was the fact that only one eye of one patient deteriorated. It is well established that, in the progress of retinopathy and maculopathy, some patients show improvement but not to the extent shown in this observational study [33].

Foveal and parafoveal cysts as seen by OCT were a feature of most of the eyes in this study. It was encouraging that for 56% or so of maculae the cysts were either reduced in size, reduced in number, or, in some cases, eliminated from the area altogether. Cyst reduction [26] or their elimination [26, 30] has been recorded in other studies that used masks to circumvent night-time dark adaption. It does seem therefore that cyst reduction and disappearance are key features of the macular improvement associated with effective light mask wear.

Intravitreal injection of anti-VEGFs results in a profound reduction in CST in sight threatened patients [34, 35]. The present Noctura 400 study did produce macular change, but of a more subtle nature than the marked but temporary macular thinning produced by anti-VEGF agents [35]. Mask wear over the year was associated with a small average thinning in 8 of 9 zones which was significant in 4 zones. Overall, 5 eyes had their best zone decreasing by upwards of 30 *μ*m, while 21 decreased by 10 *μ*m or more. The macula of only one eye thickened in all zones. Crucially, the majority of eyes remained stable in all zones at the study end. The macular pathology was not severe at the outset of the investigation; the patients being selected on the basis of their not requiring standard interventional treatment at the time of recruitment. As a result, zone improvement in a proportion of eyes was encouraging, while the lack of progression in all but one eye was reassuring.

It was a feature of the present investigation that, by study exit, the patients' eyes exhibited a highly significant improvement in VA. Further analysis showed that 47 of the 48 had some level of functional visual benefit. Although for 42 eyes, the improvement was greater than 5 letters (87.5%) and resulted in a mean gain of 12.4 letters for best VA. Although there were no improvements in VA at all in CLEOPATRA [28], that was not the case for the other two studies. INSIGHT [26] showed a small but significant improvement in BCVA but strangely this was in the young and elderly “normals” not in their DMO group. It seems that visual improvement in both the DMO eye and the normal contralateral eye of patients wearing this bilateral light treatment mask has been reported to the Noctura 400 manufacturers (PolyPhotonix) on numerous occasions (Personal Communication Prof. Ian Grierson, Chief Clinical and Scientific Officer, PPX). Furthermore, in the Prague study [27], the deteriorating trend in VA seen over an average 3 year period prior to recruitment was reversed during the 6 months of mask wear. Admittedly, these studies [26, 27] did not gain the VA improvements seen here but were for shorter duration and entirely different subject groups with respect to disease status.

We believe that our functional and anatomic successes are down to good mask wear. On average our patients wore their Noctura 400 for around 5.4 hours per night. This study is one of 4 studies to use the Noctura 400, so how do they compare? The INSIGHT safety trial had a duration of three months and the diabetics in the study also wore the mask for around 5.4 hours on average [26]. On the other hand, the 6-month Prague trial [27] was slightly less by averaging 5.0 hours, while in complete contrast to the others, mask wear in the CLEOPATRA phase III trial [28] was worryingly low, possibly around 1.5 hours of average use per night. We cannot from their available data make a precise calculation in terms of hours per day per patient but we can get some idea. It is clear that there were substantial mask wear problems even at the first 4-month assessment because 55% of Noctura 400 wearers did not reach 4 hours per night and at the study end that had risen to an alarming 74%. Clearly, a median somewhere around 3 hours is the best they could have recorded early in their trial with substantial drop-off by the study end to around half that figure.

It seems that 5-hour mask wear per night or more is achievable over 3 months, [26] 6 months [27], and 1 year (present study) in single-centre investigations with well-established patient communication. Patient adherence to long-term use of any device or medication is always a challenge [36]. Key features include thoroughly educating the patient in the workings and science behind their treatment so they are comfortable with it, establishing a strong patient/health professional relationship with good two-way communication, nurturing trust is crucial, and the patient needs an outlet to share their illness experience, become enthusiastic, and engage fully with their therapy [36]. These and other issues were considered in previous studies [26, 27] with the learning applied in this present study.

Moreover, there are additional challenges to engage with as the home treatment branches out to multiple centres but we believe that key factors in gaining decent mask wear are to establish patient understanding of their treatment, enthusiasm for adherence, and a good rapport between home and clinic. In resource-strapped hospital settings, such engagement and support may initially seem to be off-putting but our study was conducted through the year in a “Real-World” setting with no extra clinical resources. Thus, we believe that our mask wear experience can be replicated in multiple settings in both the UK and abroad and remain confident that the CLEOPATRA experience [28] was an aberration but that issue still needs to be fully resolved.

We have firm data for mask wear but compliance has been calculated differently in the various studies. Two investigations calculated compliance as a percentage of the mask's available light dose [26, 28] or just simply reported the average hours of mask wear against reported sleep duration for the elderly [27]. As the 8-hour light dose was an arbitrary setting in the pod of Noctura 400, it only would make physiological sense as a component of compliance calculation if our diabetic patients were likely on average to sleep 8 hours. Except for a few, that is highly unlikely given that elderly often sleep less [37] and diabetics are notoriously poor sleepers on the whole [38]. Total compliance is when a light mask completely eliminates sleep-associated dark adaption [25]. Therefore if a patient sleeps on average 7 hours and wears the operating mask 6 hours, the patient is 85.7% compliant not 6/8 hours (75.0%) compliant.

Kuchynka et al. in the Prague study [27] acknowledged the issue by selecting an estimated average sleep time value for their diabetics based on the literature [38]. The rigidity of this approach once again creates its own problems as there is only one variable, the data not being patient-specific. Principally, we calculated compliance in this study based on measured night-time readings from the mask over the average of nightly actual sleep as reported by each patient. On this basis, we had initial median mask wear of around 80% for our patients that remained much the same at the study end (76%).

Major positive features of Noctura 400 treatment include the fact that it is a home-use device that can take pressure off busy ophthalmic clinics. In addition, it has a low side effect profile compared to invasive anti-VEGF and steroid implant complication issues. Even laser, in its various forms, is not without its problems. The burn scars are subject to atrophic creep and can expand to produce visual deterioration [39]. Photophobia, scotoma, narrowing of the visual field, reduced colour vision, and poor contrast perception have been associated with aggressive laser application, precipitating the development of more subtle laser approaches to treatment [40]. By way of contrast, the light mask can be considered for use at the earliest stages of disease development. However, effective mask wear involves reasonably constant nightly use that is not within the capabilities of all patients. It is essential therefore to identify early on the minority who struggle badly and direct them towards other treatment options (it is not evident in this study but see [26, 27]) while encouraging and supporting the majority, particularly in the early stages, while they are getting used to the device. Thereafter, compliance monitoring is a crucial part of the treatment (see earlier) and the patient/doctor relationship. The present study shows that long-term use with good compliance can be achieved but this has not always been the case [28]. While sleeping, mask slippage does occur but it is an uncommon event and not reported as an adverse event in the present study. There was however one clear adverse event associated with mask wear as well as six other possibles. In previous studies [26, 27], few patients reported light-related problems (and none here) but more had issues with the structure of the fabric mask so a redesign is underway. As with any new device, the very novelty of Noctura 400 is a limitation that hopefully will be remedied by time.

The present study straddled the 7 weeks of COVID-19 lockdown in the UK and also Ramadan. Though clinic visits were cancelled due to COVID-19 restrictions, mask and patient support remained in place throughout. It is not quite clear why reported sleep dipped in this period, though in many homes, normal routines were totally disrupted; worries about the future, stress levels, mental health, altered diet, and reduced exercise all surfaced during lockdown [41]. That is to say, it is not surprising that mask compliance also dropped (median around 65%). We are encouraged that mask wear during such a disruptive period did not by any means collapse with no patient falling below 50% compliance and others remaining at the 80 and 90% mark. It shows that this home-based therapy persisted with a decent level of compliance and no patients lost treatment at a time when, in stark contrast, hospital-based services were under massive pressure. Our study highlights the need for good home treatments in these volatile times.

## 5. Conclusions

This study gave patients the Noctura 400 to wear overnight, with planned calls and patient contact during the study period. The study sample was small being limited to 25 subjects; the maculopathy improvements seen here were apparent after approximately 6 months. The VA showed an increase in the number of letters for all but 1 eye at the study exit. On OCT examination, patients showed clinically significant anatomical improvements such as a marked reduction in macular cysts. The vast majority of patients exhibited anatomic stability or improvement during the study period, eliminating their need to engage with additional in-clinic treatments. Only one patient required standard intervention due to the progression of retinopathy. We have shown that patients are capable of maintaining a high level of mask wear compliance, when properly supported, and do not exhibit the drop-off in treatment participation as commonly associated with chronic conditions [42]. Although some drop in compliance was noted, participation remained constant even when normal sleep was reduced during the COVID-19 pandemic first lockdown. The median level of mask wear compliance over time during the whole study started at 80% and ended at 76%. In addition to the good compliance outcomes, statistically relevant clinical outcomes were also observed. In these days of limited hospital resources and unprecedented health challenges, there is a worldwide need for inexpensive home-based treatments to help maintain continuity of care and provide new therapeutic directions to vulnerable diabetics, to combat DR and DMO.

## Figures and Tables

**Table 1:** The age, sex distribution, HbA1c levels at each visit, type of diabetes, and diabetes control measures for our 26 patients of which 24 completed the study.

**Age in years at baseline**: median (range) for our 26 recruited patients		62.5 (51.5–73.0)
**Sex (m/f)**		(21 male–5 female)
**HbA1c:** median (range) mmol/mol	Baseline	63 (52–74)
	Visit 2	63 (52–73.5)
	Visit 3	63 (52–73.5)
	Visit 4	60 (54–69)
	Visit 5	58 (54–63.5)
	Prelockdown	63 (55–73)
	Lockdown	56 (52–66)
**Type I diabetes**		11.5% (3/26)
**Type II diabetes**		88.5% (23/26)
**Diabetes control**	Diet-controlled	1/26 (3.85%)
	On insulin	12/26 (46.15%)
	On tablets	13/26 (50%)

**Table 2:** Median and range of percentage compliance for the masks returned at each visit based on reported sleep hours and measured mask wear.

Mask collection point	Median (range) of % mask wear to sleep
Visit 2	80.06 (64.54–89.97)
Visit 3	79.43 (65.59–91.17)
Visit 4	73.14 (57.72–88.64)
Visit 5	76.61 (53.96–94.12)
Prelockdown	77.58 (61.39–94.11)
Lockdown	65.39 (50.69–92.61)

**Table 3:** The mean and median OCT measurements for the maculae of the 24 patients who completed the study. The table includes values for each of the 9 macular zones and a best improvement for all zones determination. Parametric (paired *t*-test) and nonparametric (signed-rank test) stats testing have been applied.

Outcome	Mean (SD) difference (last-first visit)	Median (IQR) difference (last-first visit)	*p* value paired *t*-test	*p* value signed-rank test
Volume of macula (*μ*m^2^)	−0.08 (0.15)	−0.07 (−0.13–0.01)	0.0023	<0.0001
Central maximum thickness	4.77 (32.44)	−2.5 (−9.5–8.5)	0.2087	0.6674
Zone 1: central subfield thickness (*μ*m)	1.27 (23.05)	−2 (−8–7)	0.5102	0.42
Zone 2: inner nasal subfield thickness (*μ*m)	−2.25 (6.26)	−2 (−5.5–1)	0.048	0.0018
Zone 3: inner superior subfield thickness (*μ*m)	−2.17 (11.71)	−2 (−5–1)	0.3529	0.003
Zone 4: inner temporal subfield thickness (*μ*m)	−2.79 (13.17)	−2.5 (−5–2)	0.1419	0.0141
Zone 5: inner inferior subfield thickness (*μ*m)	−2.58 (11.72)	−2 (−5–0)	0.1481	0.0045
Zone 6: outer nasal subfield thickness (*μ*m)	−0.27 (14.92)	−2 (−4–0)	0.8811	0.0014
Zone 7: outer superior subfield thickness (*μ*m)	−3.52 (11.21)	−2 (−5–1)	0.047	0.0015
Zone 8: outer temporal subfield thickness (*μ*m)	−1.79 (5.94)	−1 (−4–1)	0.05	0.021
Zone 9: outer inferior subfield thickness (*μ*m)	−2.6 (4.91)	−3 (−5–0.5)	0.002	0.0001
Best of all zones (*μ*m)	−13.26 (14.17)	−8.5 (−16–4)	<0.0001	<0.0001
Best of inner ring zones (*μ*m)	−10.52 (13.82)	−5.5 (−15–3)	<0.0001	<0.0001
Best of outer ring zones (*μ*m)	−7.11 (9.52)	−5 (−8–2)	<0.0001	<0.0001

**Table 4:** VA and contrast sensitivity determinations that show differences between baseline and exit. While there is no significant improvement in contrast sensitivity, there is a highly significant letters' improvement in VA in both parametric and nonparametric testing.

Outcome	Mean (SD) difference (last-first visit)	Median (IQR) difference (last-first visit)	*p*value paired *t*-test	*p*value signed-rank test
Visual acuity	13.19 (8.03)	15 (10–16.5)	<0.0001	<0.0001
Visual acuity with pinhole	13.52 (7.01)	15 (10.5–18)	<0.0001	<0.0001
Best visual acuity	12.38 (7.18)	14 (9–16.5)	<0.0001	<0.0001
Contrast sensitivity	−0.003 (0.13)	0 (−0.15–0)	1	0.8902

## Data Availability

Raw data were generated at Ashford and St. Peter's Hospitals NHS Foundation Trust. The derived data supporting the findings of this study are available from the corresponding author upon request.
